# *In Vivo* Delivery Systems for Therapeutic Genome Editing

**DOI:** 10.3390/ijms17050626

**Published:** 2016-04-27

**Authors:** Luyao Wang, Fangfei Li, Lei Dang, Chao Liang, Chao Wang, Bing He, Jin Liu, Defang Li, Xiaohao Wu, Xuegong Xu, Aiping Lu, Ge Zhang

**Affiliations:** 1 Institute for Advancing Translational Medicine in Bone & Joint Diseases, School of Chinese Medicine, Hong Kong Baptist University, Hong Kong 00852, China; luyaoben@126.com (L.W.); fayebalaba@live.com (F.L.); danglei_hkbu@163.com (L.D.); liangchao512@163.com (C.L.); wangchao@hkbu.edu.hk (C.W.); hebinghb@gmail.com (B.H.); liujin_hkbu@163.com (J.L.); lidefang@163.com (D.L.); wxho0606@163.com (X.W.); 2 Central Laboratory, Zheng Zhou Hospital of Traditional Chinese Medicine, Zhengzhou 450000, China

**Keywords:** *in vivo* delivery systems, vectors, genome editing, programmable nucleases

## Abstract

Therapeutic genome editing technology has been widely used as a powerful tool for directly correcting genetic mutations in target pathological tissues and cells to cure of diseases. The modification of specific genomic sequences can be achieved by utilizing programmable nucleases, such as Meganucleases, zinc finger nucleases (ZFNs), transcription activator-like effector nucleases (TALENs), and the clustered regularly-interspaced short palindromic repeat-associated nuclease Cas9 (CRISPR/Cas9). However, given the properties, such as large size, negative charge, low membrane penetrating ability, as well as weak tolerance for serum, and low endosomal escape, of these nucleases genome editing cannot be successfully applied unless *in vivo* delivery of related programmable nucleases into target organisms or cells is achieved. Here, we look back at delivery strategies having been used in the *in vivo* delivery of three main genome editing nucleases, followed by methodologies currently undergoing testing in clinical trials, and potential delivery strategies provided by analyzing characteristics of nucleases and commonly used vectors.

## 1. Introduction

**Figure 1:**
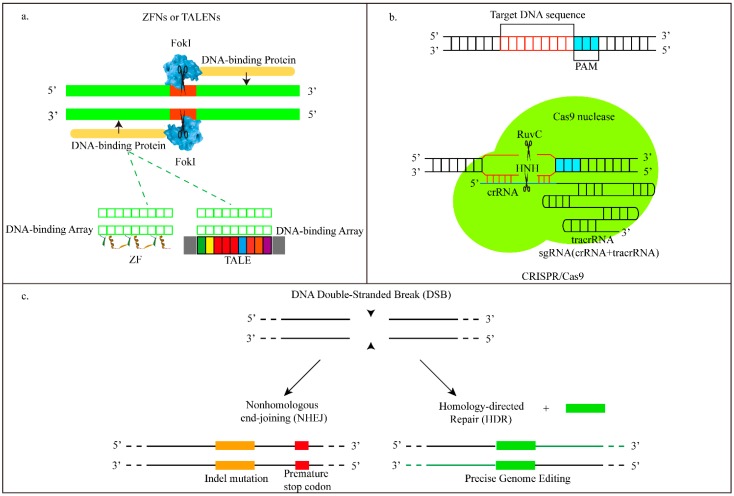
Endogenous DNA repair and three genome editing tools. (**a**) Zinc finger (ZF) and transcription activator-like effector (TALEs) proteins are DNA-binding domains which can be modularly combined to bind targeted sequences. ZF and TALE domains can separately recognize three and one bp of DNA. These site-specific nucleases are the fusion of DNA-binding domains and *Fok*I endonucleases; (**b**) Cas9 nucleases in the clustered regularly-interspaced short palindromic repeat-associated nuclease Cas9 (CRISPR/Cas9) system target specific DNA sequences with the guide aid of sgRNA, then directly achieve base-pairing with target sequences. The binding of the Proto-spacer adjacent motifs (PAM, blue) downstream of target sites aids to directing Cas9-mediated double-stranded break (DSB); and (**c**) DNA DSBs can be typically repaired via non-homologous end-joining (NHEJ) or homology-directed repair (HDR), from reference [15].

Therapeutic genome editing is mediated by sequence-specific targeting nucleases, also known as programmable nucleases. To date there are three major classes of programmable nucleases: zinc finger nucleases (ZFNs), transcription activator-like effector nucleases (TALENs), and the clustered regularly-interspaced short palindromic repeat-associated nuclease Cas9 (CRISPR/Cas9) [1]. They opened up the possibility of achieving, directly, correcting genetic mutations in target pathological tissues and cells to cure diseases (Figure 1). Compared to the other two powerful genetic therapeutic technologies, gene therapy and RNA interference, genome editing technologies enable more precise gene modulation by inducing DNA DSBs at specific genomic site via designing targeted nucleases with site-specific DNA binding domains [1,2]. ZFNs and TALENs, sharing the same *FokI*-derived nuclease domain, employ different DNA binding arrays: zinc finger arrays, and TAL effector repeats [3,4]. CRISPR/Cas9 employs sgRNA to induce site-specific genome editing in target cells with high frequency [5]. However, inefficient modification of target loci often results from inefficient delivery, making cells lack robust delivery platforms. Clinical applications of these programmable nuclease complexes are hampered by their inability to reach the intended target tissue, cross the cell membrane, and exert their therapeutic activities *in vivo*.

**Figure 2:**
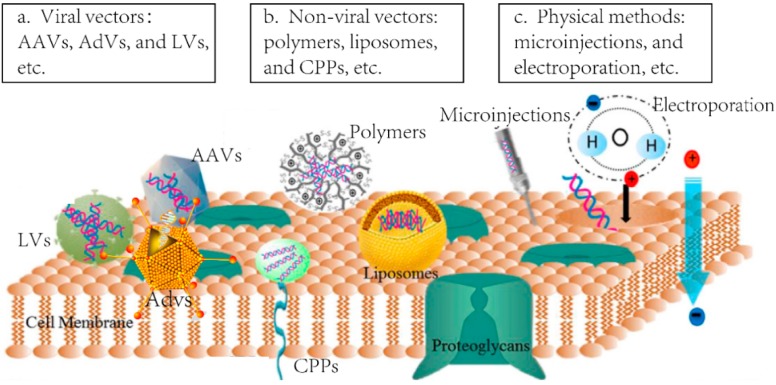
Current techniques used for gene delivery. (**a**) Viral vectors containing adeno-associated virus vectors (AAVs), adenovirus vectors (AdVs), and lentivirus vectors (LVs), *etc.*; (**b**) non-viral vectors containing polymers (e.g., polyethylenimine—PEI, poly(L-lysine)—PLL), liposomes (e.g., 1,2-dioleoyl-3-trimethylammonium-propane—DOTAP, cholesterol), and cell-penetrating peptides (CPPs), *etc.*; and (**c**) physical methods containing microinjections, and electroporation, *etc.*, from reference [16].

Both physical methods and delivery vectors are employed in the delivery of nuclease-based genome editing system (Figure 2). In physical methods, such as microinjection, electroporation, ballistic delivery, and laser, physical energy is used for cell entry [6]. Nevertheless, physical methods are more suitable for *in vitro* delivery. Vectors, like viral vectors and non-viral vectors, can encapsulate the plasmid or mRNA of these programmable nucleases or nuclease proteins, and carry them into target tissues or cells without degradation. Development of safe and efficient delivery vectors becomes more and more significant. To date, vectors used for gene-based systemic delivery in clinical trials include viral vectors [7] such as lentivirus vectors (LVs), adenovirus vectors (AdVs), adeno-associated virus vectors (AAVs) and herpes simplex-1 virus vectors (HSV-1s), and non-viral vectors [8] such as lipid nanoparticles (LNPs), liposome, polymers, and conjugates, as well as some novel ones such as cell-derived membrane vesicles (CMVs) [9]. Being exploited as a “Trojan Horse” for genome therapeutic technologies, viral vectors whose parental wild-type viruses are rearranged to hinder replication or generation of infectious virions. On the contrary, their ability of delivery nucleic acids for reaching and penetrating specific target cells and expressing genetic information in these cells is maintained [10]. Ideal virus-based vectors for therapeutic genome editing can avoid the expression of viral genes and consequently avert the toxicity. However, even being rearranged, the perishing adverse effects of viral vectors still exist. A clinical trial of applying the gene for ornithine transcarbamylase (OTC), delivered by the second-generation of E1 and E4 deleted AdVs, on the liver of the patient (Gelsinger) who suffered from a partial insufficiency of OTC caused the patient’s death in 1999. There were also some similar accidents, such as the retroviral vector inducing a lymphoproliferative disorder (2002–2003) [7]. Hence, the toxicity of viral vectors is a major issue of concern when applying viral vectors in genome editing therapy.

Compared to viral vectors, non-viral vectors are a type of burgeoning vectors. They have the potential to address limitations of viral vectors such as immunogenicity [11], carcinogenesis [12], and limited encapsulating capacity [7]. Nevertheless, only a few non-viral vectors mediated gene therapy strategies have so far been applied in clinical trial owing to the low *in vivo* delivery efficiency of non-viral vectors relative to viral vectors. Additionally, several recently-reported non-viral vectors under clinical evaluation in 2014 [8], only one non-viral vector of a total 2210 vectors was reported in the statistics on the topic of “Vectors Used in Gene Therapy Clinical Trials”, while 66.4% of vectors applied in gene therapy clinical trials were viral vectors [13]. Now this drawback is overcome by modifying raw materials of non-viral vectors and improving engineering recipes. For example, in 2015, Chunyang Sun’s group reported their novel study on an established pH_e_ (dysregulated pH scale in tumor) sensitive micelleplex siRNA delivery system whose corresponding nanoparticles (Dm-NP) might undergo several modifications, and the results showed that the novel delivery system they produced can specifically target cancer cell [14]. Furthermore, many other types of vectors made from neoteric materials, such as the endogenous carriers, cell-derived membrane vesicles (CMVs), are also extensively studied [9].

In this review, we summarized current strategies of *in vivo* delivery of three main genome editing nucleases, followed by methodologies undergoing evaluation in clinical trials, as well as suggestions on potential delivery strategies by analyzing characteristics of nucleases and commonly-used vectors (Table 1). Considering the clinical translation, promising vectors under clinical investigations are highlighted.

## 2. *In Vivo* Delivery Systems for Zinc Finger Nucleases (ZFNs) and Their Expression Cassette

Zinc finger nucleases (ZFNs) are the first sequence-specific designer nucleases for gene editing. ZFNs consist of a DNA recognition domain (Zinc finger proteins, ZFPs) which is composed of 3–6 Cys_2_-His_2_ zinc fingers and a non-specific DNA cleavage domain (derived from *Fok*I endonucleases) via the C-terminal of the cleavage domain [17,18]. Each zinc finger domain of ZFPs can recognize 3- to 4-bp DNA arrays via a single α-helix, and several tandem zinc finger domains can typically recognize and bind to DNA sequences with the length of multiple of three, normally 9- to 18-bp with high specificity [19]. Then DNA cleavage domains in ZFNs can cut the DNA sequence recognized by the ZFPs [19]. By this mechanism, ZFNs-based genome editing technologies are used to alter genomic sequences to correct a mutation or create a mutation.

Actually, before developing ZFNs, homing endonucleases (HEs), the natural meganucleases, have been utilized for genome editing for more than 15 years [20,21,22,23,24,25,26,27]. Compared to HEs, ZFNs-based genome editing technologies are more effective. A representative example is a mutation created by ZFPs-based genome editing in C–C chemokine receptor type 5 (CCR5) making cells resistant to HIV infection [28]. Nevertheless, unwanted off-target effects cannot be avoided because the wild-type *Fok*I enzyme can still cleave the non-target DNA even when only one monomer binds to this DNA sequence [29]. Efforts to improve the specificity of ZFNs include the development of heterodimer cleavage domains, optimization of the spacer requirements between two target DNA sections via linker design, enhancement of cleavage activity through directed evolution of the *Fok*I nuclease, as well as investigation of a proper and efficient *in vivo* delivery system. Commonly used *in vivo* delivery vectors for ZFNs system are presented as follows:

### 2.1. Viral Delivery

The thought of using viruses as vectors to deliver a package of genetic information to achieve gene therapy was firstly successfully demonstrated in 1990, when the purified, healthy T-lymphoid cells of a patient suffered from both adenosine deaminase deficiency (ADA) and immunodeficiency (SCID) were redelivered in the body via a retroviral vector. This therapy repaired the immune system of the patient. [30]. Two main parts of the virus life cycle are infection and replication. In the infection step, virus enter into target cells after recognition, then release the viral genome for replication. In the replication step, progeny virions are released outside cells after synthesizing the viral genome copies in cells. Then, new infection steps in nearby cells or circulation begin [10]. Based on this mechanism, viruses are used as vectors to encode and deliver genome editing programmable nucleases to target tissues or cells, and achieve genome editing therapy.

#### 2.1.1. Adenoviral Vectors (AdVs)

Adenoviruses (Ad), belonging to the family of *Adenoviridae*, were firstly discovered in 1953 and isolated from cultures of human adenoid tissues. The first-generation adenoviral vectors (AdVs) are obtained by substituting the gene E1 (3.15 kb), or the E1 and E3 (3.1 kb), followed by the second-generation AdVs, which lack more than two early genes, including E2 and E4. Compared to the first generation, the second-generation AdVs provide an extended genome packaging capacity. However, it is limited by the inflammatory response of the host and the inability of replicating *in vivo* including a cytotoxic T-cell response [31,32]. Considering this situation, the production of safer and more efficient AdVs, targeting site-specific sequences of the disease, has therefore become a trend [33]. For systemic delivery of ZFNs, here is a good example which is against HIV infection and has entered clinical trials: the expression unit coding the right and left ZFNs is inserted into a serotype 5 AdV pseudotyped with serotype 35 fiber (AdV5/35). The AdV/ZFNs system is designed to repair the autologous CD4^+^ helper T cells from HIV infected patients [34,35].

A shortcoming of AdVs: as the receptor of Ad on cell, the Coxsackie-adenovirus receptor (CAR) level determines the delivery efficiency of AdVs. For instance, the expression level of CAR in cancer cells is mutable probably negative correlation to the malignancy level of the tumor, which means the CAR is rare in cancer cells. Therefore, investigating the targeted AdVs specifically for cancer cells is playing an emerging role in anti-tumor research via genome editing technology.

#### 2.1.2. Lentiviral Vectors (LVs)

Lentiviral vectors, members of the *retrovirus* family which can spontaneously penetrate the intact nuclear membrane, have now been commonly used for *in vivo* delivery in genome editing therapy. As the delivery vector of ZFNs technology, lentiviral vectors which can accommodate sequences up to around 10 kilobases (kb) theoretically allow for site-specific genome modification or addition in predefined genomic sites. However, they have been avoided because of multiple tragedies involving patient death in earlier clinical trials [36,37]. Therefore, advanced types, such as Integrase-Defective Lentiviral Vectors (IDLVs) have been heavily explored in recent years.

For instance, Angelo Lombarbo’s research group exploited one ZFNs-based genome editing system in human stem cells using IDLVs as delivery vectors [38]. The results showed up to 50% of genome addition in one dish of human cell lines, and 5% of genome addition in human embryonic stem cell lines. However, the efficiency of IDLVs may vary according to target tissues. For example, the transduction efficiency of hepatocytes in mice with IDLVs is less than integrating LVs, while IDLVs can achieve highly-effective gene transfer and expression in mice muscle *in vivo* [37,39].

#### 2.1.3. Adeno-Associated Viruses Vectors (AAVs)

Adeno-associated viruses vectors (AAVs), the commonly used delivery vector in genome editing technologies for its potential site-specific integration ability and low immunogenic characteristics, are icosahedral non-enveloped viruses in the *Dependovirus* genus of *Parvoviridae* family. The major limitation of AAV vectors system is their relatively small genome size (around 4.7 kb), which restricts the genome engineering nuclease complexes they can carry up. However, with careful design, sequences for expressing ZFNs (each sequence is around 1 kb) and an optional donor DNA template can be encapsulated well by AAV vectors [10].

The representative type of AAV vectors system is the modified one: recombinant vectors based on adeno-associated viruses (rAAV) which have been heavily explored in recent years [40]. One ZFNs-mediated gene editing drug being designed to be carried via rAAV vector to stimulate gene targeting was reported in 2013 [41]. In this research, rAAV/ZFN particles with DNA restoration substrate of 750 nucleotides were generated. The delivery efficiencies of rAAV to both human HEK-293 cell lines “293/GFP” and primary mouse fibroblasts were estimated *in vitro*, as well as in mouse and dog models *in vivo*. Results generally showed that increased AAV-ZFN2/1/donor vectors leaded to better targeting effect. Additionally, multiple other investigations on AAV/ZFNs-mediated genome editing have been obtained the positive results in animal model *in vivo* experiments [42,43,44,45]. For instance, there is one study on the *in vivo* modification of a humanized mouse model of hemophilia B: in this study, the introduction of AAV on ZFNs achieved up to 45% site-specific cleavage of the hepatocytes [46].

### 2.2. Non-Viral Vectors

Non-viral vectors in non-viral methodologies for genome editing can be classified into several sub-types according to their raw materials, size, or producing recipes, and so forth. For instance, they can be sorted into lipid-based vectors, and polymer-based vectors, and the like, when raw materials are lipids, polymer, *etc*. Lipid-based vectors can then be classified as lipid nanoparticles (LNPs) and liposomes, and so on, according to various producing recipes and sizes. Liposomes, polymersomes, LNPs, lipoplexes, polyplexes, dendrimers, and cell-penetrating peptides (CPPs) are commonly used to deliver genes.

Compared to immunogenic viral vectors, whose capacities are relatively small, non-viral vector-mediated strategies possess advantages of low toxicity, high gene transfection efficiency, large loading capacity and easy producing procedures. However, the low transfection efficiency plus the poor transgene expression impede the clinical application of non-viral vector-based delivery strategies. Therefore, to improve the transfection efficiency and transgene expression, as well as enhance the targeting efficacy and safety of delivery approaches, many studies have been done. For instance, existing liposomes, polymers and peptides-based vectors are all improved. Cationic liposomes are found to be effective for its high transfection efficiency and biocompatibility since being firstly investigated and published by Felgner’s group in the late 1980s [16,47]. However, there is rarely a report or clinical record on non-viral vector-mediated *in vivo* delivery of meganucleases for genome editing [24,25].

Given the positive charge of ZFNs particles encapsulated by cationic lipids and cationic polymers is attracted to the negative charge of cell membranes, using cationic lipid nanoparticles or cationic polymers as vectors will induce the cellular uptake of genome editing nucleases, such as ZFNs [48]. However, there is no report on the *in vivo* delivery of ZFNs by cationic lipid nanoparticles, and while some *in vitro* research is published [49,50], neither are cationic polymers.

### 2.3. Direct Delivery of ZFN Proteins

Passing through troubles which exist both in the cell membrane penetrating process of proteins caused by its low level of lipophilicity and *in vivo*, such as pH sensitivity and degradation by endogenous proteases, ZFN proteins can surprisingly penetrate cell membranes due to the positive charge of Cys_2_-His_2_ zinc finger domains [51]. The results showed that the direct delivery of ZFN proteins can disrupt the CCR5 gene in both HEK-293, HDF cells, and human CD4^+^ T cells. Even though its motion *in vivo* is unclear, the *in vivo* delivery efficiency can still be demonstrated. Moreover, compared to ZFN plasmid delivery, the off-target mutagenesis of this methodology are reduced (ClinicalTrials. Gov Identifier: NCT01044654, NCT01252641 and NCT00842634) [52]. Increasing the stability of ZFN proteins is vital to achieve the direct delivery of ZFN proteins, because ZFN proteins will be degraded completely in four hours. One of possible strategies to keep ZFN stable is to modify the lysine residue needed in the degradation [53,54].

### 2.4. Summary and Prospect

Much research has shown that ZFNs can mediate genome editing efficiently by making site-specific DSBs then inducing homology-directed repair (HDR) in target cells and the appropriate alternative and development of the *in vivo* delivery system is critical to clean barriers hampered on its clinical translation way. So far, AAVs-based ZFNs genome editing therapy *in vivo* is increasingly applied in clinical trials. AAVs/ZFNs-mediated *in vivo* gene correction in the mouse model of hemophilia is a good example [55]. AAVs, having been investigated and developed maturely as vectors via constant improvement, are commonly used in carrying ZFNs rather than TALENs or CRISPR/Cas9 for their relative small cargo size. To date, there is no AAVs/ZFNs-based genome editing therapeutic methodology being applied in clinical trials. By contrast, as the presentation above, the strategy of directly delivering ZFN proteins into target cells to achieve genome editing has been in clinical trials (Table 1). In addition, non-viral vectors, possessing advantages of low toxicity, high gene transfection efficiency, large loading capacity, and easily-produced procedures, deserve to be investigated. Filling the gap in ZFNs-based genome editing technology on non-viral vectors mediated delivery system, investigating novel and efficient non-viral vectors mediated delivery strategy is needed.

## 3. *In Vivo* Delivery Systems for Transcription Activator-Like Effector Nucleases (TALENs) and Their Expression Cassette

Transcription Activator-Like Effector Nucleases (TALENs), similar to ZFNs, also consist of two domains, a DNA-binding domain and a C-terminal *Fok*I endonuclease cleavage domain. The DNA-binding domain is composed of multiple units of 33~35 amino acid repeat arrays arranged in tandem, and it is contained in TALEs, which are natural proteins of the plant pathogenic bacteria *Xanthomonas* sp. [18]. In contrast to ZFPs, TALENs possess advanced advantages such as lower cytotoxicity [56], greater design flexibility leading to higher targeting range [57], and easier engineering. However, there is no single site-specific TALEs with long arrays due to the lack of the linkage between repeats. These TALE repeats are nearly identical in sequence. Fusing TALEs to the *Fok*I endonuclease, TALENs bind and cleave the target DNA in pairs [58].

In summary, ZFNs and TALENs both consist of an engineered DNA-binding domain and a *Fok*I nuclease domain. DNA-binding domains, engineered ZF and TALE repeats, can bind to gene sequences after being arranged in tandem. Then ZFNs and TALENs can achieve customized genome editing in target cells. To date, several *in vivo* vectors are available for delivery of TALENs by carrying their expression cassettes (more than 5 kb) which usually consist of the RNA polymerase II (Pol II) promoter, the TALE encoding sequence, the *Fok*I nuclease domain, and the polyadenylation signal [32]. AAV, the promising delivery vehicles for transgenes of ZFNs, would be not able to afford accommodating TALENs for its relatively small cargo size of ~4.7 kb [59]. Nevertheless, the other viral vectors with larger capacity can be investigated (Table 1).

### 3.1. Viral Vectors

#### 3.1.1. Lentiviral Vectors (LVs)

To deliver genome editing nucleases effectively, lentivirus have been developed for several generations from the early generation which contains the gene of cis-acting elements, such as the long terminal repeats to the second generation in which multiple non-essential accessory genes are deleted and self-inactivating vectors are established in order to avoid the unwanted generation of replication-competent lentiviral vectors (LVs) and the activation of nearby genes due to genomic integration and then the lentiviral trans-activator of transcription-independent vectors were generated in the third generation. Moreover, the central PolyPurine Tract (cPPT) for increasing vector transduction efficiency and the Woodchuck hepatitis virus post-transcriptional regulatory element (WPRE) for enhancing transgene expression levels were inserted into the recombinant LV genome [32]. However, it seems that sufficient delivery and expression of TALENS in mammalian cells via commonly-used versions of LV vector systems is not feasible, because of their potential mutagenicity caused by the deletion of complete repeats that occurred during vector production ranging from 5 to 15 repeats per TALEN [60].

#### 3.1.2. Adenoviral Vectors (AdVs)

Compare to lentiviral vectors, AdVs-mediated TALEN genome editing therapy can result in analogously high level site-specific DSBs both in transformed cells and non-transformed cells [61]. As the statement above, the first and second generation AdVs are not optimal vectors for therapeutic approaches due to their immune side effect *in vivo*, even though Holkers and colleagues have demonstrated that these AdVs can carry the TALEN expression cassette into human cells [60]. The most advanced version of AdVs are represented by high-capacity AdVs (HCAdVs) without all viral coding sequences, but only the inverted terminal repeats (ITR) and the packing signal at the 5′ end of the genome being maintained. They can be typically distinguished from the common AdVs by the reduced induction of innate and adaptive immune responses, as well as providing a total packaging capacity of up to 36 kb which offers great potential for delivery of large TALEN expression cassettes [32].

#### 3.1.3. Baculoviral Vectors (BVs)

Baculoviral vectors (BVs), derived from the insect *Autographa californica* multiple nuclear polyhedrosis virus, can accommodate 100-kb of DNA insert leading to a usage for delivery of TALEN plasmid. One research, developing a BVs-TALENs coupled with BVs-Cre/loxP genome editing tool, offers a novel strategy for TALEN-based genome editing with low genome toxicity in engineering human pluripotent stem cells. The results demonstrate that BVs-TALENs are effective in mediating genetic modification. However, TALEN-repeated sequences in a BV do not obviously affect overall TALENs activity. The present application of BVs on carrying genome editing nucleases is still limited to *ex vivo* status [57].

### 3.2. Non-Viral Vectors

#### 3.2.1. Cationic Polymer-Based Vectors

As described above, cargo size is a problem for *in vivo* delivering of TALENs. The plasmids encoding TALENs, being easier to be encapsulated, are commonly delivered into tissues or cells *in vivo* via therapeutically-efficacious cationic polymers. One vector made of cationic polymers (TurboFect^®^) is utilized to carry TALEN plasmids, targeting human papillomavirus (HPV), directly to the cervix of transgenic mice displaying HPV infection and cervical cancer. After this therapy, the tumor size is reduced, while neither off-target mutations nor signs of inflammatory response are detected [62]. Comparatively speaking, cationic lipids are rarely applied in delivering TALENs for genome editing.

#### 3.2.2. Conjugates

Refer to the methodology on direct delivery of ZFN proteins, direct delivery of TALEN proteins instead of transgenes has also been investigated. However, unlike the naturally cell-penetrating ZFNs, TALEN proteins are found to be incapable of penetrating cellular membranes when administered alone. Thus, cell-penetrating peptides (CPPs) are always utilized to promote the cell penetration of TALEN protein. Functional TAT-TALENs (YGRKKRRQRRR-TALENs), the conjugation of cell-penetrating TAT peptides and TALENs, can penetrate targeted cells and damage the genome encoding endogenous human chemokine receptor 5 (CCR5) to prevent HIV-1 from entering into cells [63]. On the contrary, this methodology is not suitable for ZFN proteins because a large amount of purification of TAT-ZFNs is so hard to achieve that the quantity cannot satisfy the analysis-required amount in cell lines. Similarly, the cell-penetrating poly-Arg9 peptides-TALEN proteins fusion enables TALEN-based genome editing of human CCR5 genes in HeLa cell lines and BMPR1A genes in HEK-293 cells, respectively [64].

### 3.3. Summary and Prospects

Due to the large size of TALENs and repetitive characteristics, it is difficult to achieve *in vivo* delivery of TALENs into targeted tissues or cells. Especially when lentiviral vectors are used for packaging, unwanted recombination events will be resulted in. Considering these above, proper delivery vectors for TALENs include high-capacity AdVs (HCAdVs), cationic polymers, and cell penetrating peptides (CPPs)-TAT, but all have not been into clinical trials yet.

## 4. *In Vivo* Delivery Systems for CRISPR/Cas9 and Their Expression Cassette

The CRISPR/Cas (Clustered regularly interspaced short palindromic repeat- associated nuclease Cas) system consists of two components: guide RNA (gRNA) and nucleases (Cas). This system is the immune system of some bacteria used for defending against foreign nucleic acids [65,66,67,68]. Various CRISPR/Cas system types achieve nucleic acid recognition in various molecular mechanisms. The most commonly used type is CRISPR/Cas9 including a conjugation of the tracrRNA (trans-activating crRNA)—crRNA (CRISPR RNA) to a single guide RNA and Cas9. Cas9 protein, derived from *Streptococcus pyogenes* or *Staphylococcus aureus*, is significantly multifunctional to defend against viral invasion [69]. CRISPR/Cas9 achieves site-specific DSBs after being led to target loci by crRNA associated with tracrRNA. Unlike ZFNs and TALENs, which both need to be particularly made according to different target loci owing to the mechanism that they are guided by protein domains, the CRISPR/Cas9 system can bind to any interested target sequences simply via altering gRNA and achieve DSBs [70].

However, the large size of the CRISPR/Cas system presents an obstacle for its delivery *in vivo*. The size of the common-type spCas9 (from the bacterial species *Streptococcus pyogenes*) is ~4.2 kb, which states a challenge to package it while AAV, whose cargo size is ~4.5 kb, is utilized as a vector [71]. Even though the cargo size of non-viral vectors like cationic liposome or PEI can be made up to much larger than 4.5 kb, the endothelial gap size limitation of blood vessels and so forth also exist to hamper the delivery of the CRISPR/Cas system to targeted tissues for genome editing therapy *in vivo*. The limited cargo size of vectors leaves little space for engineered expression or control domains. To solve this kind of problem, a slightly smaller type saCas9 (from species of *Staphylococcus aureus*, ~3.2 kb), which is still efficient and broadly-targeting, has recently been investigated [71]. On these bases, delivery vectors are developed as follows:

### 4.1. Viral Vectors

Until now, AAV has been well-developed and utilized in delivering the CRISPR/Cas system into targeted issues and cells such as brain cells via stereotactic injection in adult mouse brains [72], as well as skeletal and cardiac muscle cells by tibialis anterior muscle injection in *mdx* mouse muscle, and so on [58]. In a mammalian brain study, SpCas9 and gRNAs are delivered by separate AAVs respectively, with shortened neuron-specific promoters *in vivo* in order to target and edit both single (Mecp2) and multiple (Dnmt1, Dnmt3a, and Dnmt3b) genes in the adult mouse brain [72]. On the basis of the significant finding on the smaller Cas9 enzyme—saCas9 [71], three newer studies focus on *in vivo* CRISPR/Cas9-based genome editing via delivering a saCas9 plasmid or Cas9 mRNA into the *mdx* mouse model by AAV8 or AAV9 to cure Duchenne Muscular Dystrophy (DMD) [59,73,74]. For instance, Mohammadsharif Tabebordbar and collaborators developed a direct genome editing approach based on the CRISPR/saCas9-AAV system and applied it to the *mdx* mouse model to introduce exon deletion and dystrophin expression restoration, and test the efficiency of different Cas9 serotypes to obtain the most efficient one. Results showed that the AAV-CRISPR system partly restored the muscle functional deficiency in *mdx* mouse muscle [73]. Moreover, many other investigators are also devoted to develop the AAVs delivery system for targeting CRISPR/Cas to different interesting tissues or cells *in vivo*, such as the central nervous system, cancer cells, and so forth [71,75,76,77,78,79].

However, the small cargo size continues to hamper AAVs as delivery vectors of the CRISPR/Cas system. To accommodate larger payloads, other viral vectors with larger capacities, such as adenoviral and lentiviral vectors, have also been investigated by various researchers for the CRISPR/Cas9 system delivery [80].

### 4.2. Non-Viral Vectors

#### 4.2.1. Cationic Lipid-Based Vectors

The anionic nature of both Cas9 protein itself, and the plasmid or mRNA encoding it, as well as gRNA, allows the integration of Cas9-gRNA complexes into the cationic liposome, which means the CRISPR/Cas9 genome editing system could be delivered via cationic liposomes. One successful case is on the Cas9 protein/sgRNA system which is encapsulated and carried to hair cells by cationic liposomes to achieve genome editing therapy in the mouse inner ear *in vivo* [81]. This approach results in up to 80% gene modification in cultured hair cells. However, it is injected directly into the inner ear of mice, but not intravenous injection which is more efficient and closer to the clinical injection approach, but more challenged. There is still a long way to go to achieve the *in vivo* delivery of the CRISPR/Cas system.

Up to now, to solve the encapsulating and *in vivo* delivery problem on the large-size CRISPR/Cas9 system, the methodology of separately encapsulating Cas9 plasmids (or Cas9 mRNA) and gRNA in two vectors, which calls for smaller cargo size, is involved into *in vivo* genome editing technology in addition to senior strategies, such as carrying the saCas9/sgRNA system [74].

#### 4.2.2. Cationic Polymer-Based Vectors

Similar to the mechanism of cationic liposomes, the cationic nature of cationic polymer nanoparticles also provides themselves the feasibility for delivery of the CRISPR/Cas9 system. Polyethyleneimine (PEI), the most common cationic polymer, consists of a secondary amine which can help to prevent DNA and endosomal escape through the proposed proton sponge effect. In addition, the structural properties, degree of branched or linearity, and molecular weight also play a vital role in deciding the transfection efficiency and toxicity of PEI [82]. Here is one example in which PEI-CRISPR/Cas9-mediated somatic genome disruption technology is established to achieve the *in vivo* targeting of tumor suppressor genes (TSGs) and delete single (Ptch1) and multiple (Trp53, Pten, Nf1) genes in the mouse brain. Nevertheless, the low spatial accuracy of transgene expression leading to low viability became its drawback [83]. Furthermore, the branching degree and the molecular weight of PEI are strongly influential to its transfection efficacy and toxicity. In details, PEI with a higher molecular weight, higher branching degree, and bigger cationic charge can form small polyplex with high enzymatic stability but accompany with the unwanted increase in the cytotoxicity. On the contrary, lower molar mass leads to less cytotoxicity, but also less efficiency [84].

To improve the transfection efficiency but decrease the cytotoxicity, many modifications of PEI are developed and some of them have been used in clinical trials. The IL-12-PEG-PEI-cholesterol lipopolymer treating persistent and recrudescent epithelial ovarian, and primary peritoneal cancer, and so on is a realizable example [84,85]. Moreover, another interesting research focuses on carrying the CRISPR/Cas9 system using 7C1 nanoparticles which are synthesized via blending C15 epoxide-terminated lipids with the low molecular weight PEI. This methodology improves the gene mutation efficiency in the pulmonary and the cardiovascular endothelium, as well as reproduces Cre-dependent Cas9 mice [77].

#### 4.2.3. Conjugation

Similar to TALENs, not only can encapsulating achieve to carry and deliver CRISPR/Cas9 system *in vivo*, conjugating is also available. Suresh Ramakrishna and colleagues present cell-penetrating peptides (CPP)-based delivery methodology in which the gRNA is complexed with CPP to form condensed and positively-charged nanoparticles, while the Cas9 protein is fused with CPP by the thioether bond. This induces efficient genome editing with reduced off-target mutations in human cells, like embryonic stem cells, HEK-293T cells, and HeLa cells, and so forth. In addition, this strategy does not need extra transfection reagents, and directly carries gRNA and Cas9 protein to reduce off-target effects [86].

### 4.3. Combined Viral and Non-Viral Delivery

Recently, a novel *in vivo* delivery approaches for CRISPR/Cas9 was established by Xue and Anderson’s laboratory. They combined viral and non-viral vectors, respectively carried mRNA of Cas9 via lipid-based vectors, and generated AAVs encoding sgRNA with the repair template. They then applied this genome editing approach to a mouse model of human hereditary tyrosinemia to induce the repair of the disease gene. The repair efficiency was more than 6% in hepatic cell lines. In addition, fumarylacetoacetate hydrolase (Fah)-positive hepatic cell lines were generated [87]. A HDR template was involved into the genome editing system and recombination of segmental HDR-mediated donor templates may happen in gene repair. Hence, the targeted *in vivo* delivery of a HDR-mediated donor template to specific genomic locus will also be crucial to therapeutic genome editing [88,89].

### 4.4. Summary and Prospects

The CRISPR/Cas9 system can be delivered *in vivo* in the form of Cas9 proteins/gRNA, plasmids encoding Cas9/gRNA, or mRNA encoding Cas9/gRNA. Plasmids with gRNA is commonly used due to the difficulty of delivering the other two forms: the size of proteins is too large to deliver *in vivo* followed by the immune response of proteins in body, while the cost of producing mRNA is too high to afford on. On basic of this, presently successful *in vivo* delivery systems for CRISPR/Cas9 include AAV, cationic liposomes, PEI, and CPP. In addition, hydrodynamic injection is also applied to assist in achieving carrying a large-size CRISPR/Cas9 system into mouse hepatic tissue *in vivo* [89,90]. Physical means such as electroporation and microinjection, facilitating uptake of plasmids into target cells, show the potential. Some electroporation-induced genetic modification *in vitro*, *in vivo*, and *in utero* are successful examples [91]. Moreover, a formulation comprising non-ionic poloxamer CRL 1005 and cationic surfactant benzalkonium chloride has entered into Phase II/III clinical study [92] (NCT01877655, NCT01903928, NCT00285259).

## 5. Conclusions and Future Perspectives

The presently-existing three programmable nuclease systems all offer pros and cons (Table 2). In conclusion, ZFNs show relatively weaker cleavage of chromosomal DNA, while TALENs possess almost 100% cleavage efficiency in mammalian cell lines [93]. Nevertheless, the high mutation rates of TALENs caused by mismatched dimer formation cannot be ignored [94]. In addition, unlike ZFNs being effective on both deletion and insertion, it is easier for TALENs to induce deletion than insertion [95]. By contrast, various Cas nucleases can be guided to different target gene sequences easily via just altering the gRNA, and the customized gRNA can be synthesized in one cloning procedure. Each coin has two sides, the convenience of the Cas9/sgRNA system of CRISPR is also the main difficult part of encapsulating and delivery *in vivo*. These disadvantages, above all, hampered the transition of nuclease-based genome editing technologies from basic studies to clinical trials (Table 3).

To solve this challenge, establishing effective *in vivo* delivery systems is crucial to the clinical transition of genome editing therapy. Up to now, these nucleases have been mainly delivered *in vivo* by viral vectors and non-viral vectors. Among them, viral vectors possess superiorities of not causing potential problems of insertional mutagenesis and having high transduction efficiency, as well as long-term fine genome expression. However, limitations still exist, such as immunogenicity, expense, and small cargo size. The well-developed viral vectors, such as AAVs and LVs, which both do well in carrying ZFNs (smaller size) into target cells, are limited in the delivery of TALENs and CRISPR/Cas9. In details, only one TALEN element with one tiny promoter sequence can be encapsulated into AAVs, and it may cause undesirable rearrangements of TALEN’s high homologous sequences in cells while using LVs as their delivery vectors [61,96]. For CRISPR/Cas9, only the saCas9/sgRNA system can fit into AAVs. Even though there are some reports on integrating LVs-encapsulated Cas9/sgRNA-based genome editing therapies with nearly 100% mutation efficiencies at target loci, the risk of exacerbating off-targeting caused by this method limits their development [97,98]. On the contrary, non-viral vectors offer an excellent ability to take large size genomic drugs and are easily generated. Nevertheless, their clinical applications are limited due to the high toxicity of raw materials, low transfection efficiency, and poor target specificity.

In terms of delivery methods for these gene editing technologies, the current literature indicates that much can be learned from the large body of knowledge obtained from nucleic acid delivery, protein delivery, and technologies developed for specific routes of administration [99]. In addition, the improvement in nanotechnology of pharmaceutics and material science pushes the emergence of novel synthetic vectors with targeting abilities and superior physicochemical characteristics. For example, mesoporous silicon particles [100] and cationic nanoemulsions can be utilized as vectors for plasmid [101]. All of these contribute to the development of *in vivo* delivery systems for genome editing technologies.

Many investigators have established novel delivery systems. For instance, Zhi Yao He, *et al.* developed folate-linked lipoplexes to deliver short hairpin RNA (shRNA) to target sites to cure ovarian cancer [102]. This can also be applied to the *in vivo* delivery of the CRISPR/Cas9 genome editing system. Moreover, long term research highlights the importance of optimizing the Cas9/gRNA system to achieve targeted *in vivo* delivery and avoid off-target mutations [103]. Three AAV-mediated delivery systems succeeded in delivering CRISPR/Cas9 genome editing system into the muscle of *mdx* mice *in vivo*. However, due to the size requirement of AAV (<4.7 kb), the application of this delivery system is still limited. Considering the situation above, delivery systems which possess large loading capacity for CRISPR/Cas9 plasmids with tissue selectivity would be preferred. Hence, delivery systems for plasmids, having entered into clinical trials including cationic liposomes, cationic polymers, and lipopolymers which are the synthetic complexes of lipids and polymers, are theoretically available for the delivery of genome editing systems (NCT01455389, NCT00595088, NCT01118052, *etc.*, Table 1) [8].

## Figures and Tables

**Table 1:** Summary form of typical *in vivo* delivery systems and candidates for genome editing nucleases and their expression cassette.

Typical Delivery Systems		Assessment		Genome Editing Nuclease	Clinical Trials			
		Advantages	Disadvantages		Phase	Status	Clinical Trials. Gov Identifier	Reference
**AAVs**		High efficiency	Low packaging capacity, cost high	ZFNs, CRISPR/Cas9	–	–	–	[59,73,74,104]
**AdVs**		Low off-target mutagenesis	Immunoreactivity, high cost	ZFNs	I	Completed	NCT01044654	[49,105,106]
					II	Completed	NCT01252641	
					I	Completed	NCT00842634	
**HCAdVs**		High packaging capacity	Cell-specific targeting is difficult to achieve	TALENs	–	–	–	[32]
**CPP, e.g., TAT-TALEN proteins; CPP-Cas9 proteins**		Low off-target mutagenesis	Immunoreactivity	TALENs, CRISPR/Cas9	–	–	–	[63,64,86]
**Candidates for delivering plasmids of nucleases**	**DOTAP-cholesterol**	Easy to produce, large packaging capacity	Large particle size, low targeting efficiency, toxic	–	I/II	Active	NCT01455389	[8]
	**PEI**	Easy to produce, large packaging capacity	Low targeting efficiency, toxic	–	II	Active	NCT00595088	
	**PEG-PEI-Cholesterol**	Easy to produce, large packaging capacity with small particle size, low toxic	Low targeting efficiency	–	II	Active	NCT01118052	

**Table 2:** Comparison of three programmable nucleases.

Genome Editing Nucleases	DNA Targeting Specificity Determinant	Endonuclease	Average Mutation Rate	Off-Target Rate	Success Rate	Size	Cytotoxicity
**ZFNs**	Zinc-finger proteins	*Fok*I	10%	High	~24%	~1 kb × 2	Variable~high
**TALENs**	Transcription activator-like effectors	*Fok*I	20%	Low	~99%	~3 kb × 2	Low
**CRISPR/Cas9**	crRNA or sgRNA	Cas9	20%	Variable	~90%	4.2 kb (SpCas9) + 0.1 kb (sgRNA)	Low

The success efficiency is defined as the proportion of nucleases inducing mutations at frequencies more than 0.5% in HEK-293 cell lines. The average mutation efficiency is based on the frequency of non-homologous end joining (NHEJ)-mediated indels obtained at the nuclease target site [5,94,95,105,107,108].

**Table 3:** Nuclease-mediated genome editing technologies having been used in clinical trials.

Genome Editing Nucleases	Clinical Trials									
	Condition	Intervention	Target	Delivery Vector	Cell Transplantation	Company	Phase	Status	Clinical Trials. Gov Identifier	Reference
**ZFNs**	HIV Infection	Genetic: SB-728-T	CCR5 DNA	AdVs or direct delivery	Autologous CD4^+^ T cells	Sangamo Biosciences	I	Completed	NCT01044654	[49,106,109]
							II	Completed	NCT01252641	
							I	Completed	NCT00842634	
**TALENs**	Leukemia	Malignant blood cells	Genes in immune cells	N/A	Chimeric antigen receptor (CAR) 19 T cells	Great Ormond Street Hospital	N/A	N/A	N/A	[110]

## Glossary

**ZFNs**	Zinc-finger nucleases are fusions of the nonspecific DNA-cleavage domain with zinc finger DNA binding domain. The DNA binding domain can be engineered to direct the ZFN to specific desired DNA sequences, and ZFN induce targeted DSBs.
**TALENs**	Transcription activator-like effector nucleases are made by fusing a DNA cleavage domain to a TAL effector DNA binding domain. TAL effectors containing 33~35 amino acid repeat domains can be engineered to recognize base pair. Then TALENs induce targeted DNA DSBs.
**CRISPR/Cas9**	Clustered regularly interspaced short palindromic repeat- associated nuclease Cas9 is a prokaryotic immune system against foreign DNA in bacteria or archaea. They consist of a guide RNA (tracrRNA-crRNA)-being used for guiding the Cas9 nuclease to the desired DNA sequence, and Cas9 protein.
**DSB**	Double-strand break. A form of DNA damage caused by ZFN, TALEN and CRISPR/Cas9. Both DNA strands will be cleaved.
**NHEJ**	Non-homologous end joining. A DSB repair pathway which can relink broken ends together. It often leads to small insertions and deletions at the broken site.
**HDR**	Homology-directed repair. Another DSB repair pathway by which the donor molecule will be inserted at targeted sites. By HDR, single, multiple transgenes, or single nucleotide substitutions can all be inserted.
**Indel—insertion/deletion**	A molecular biology term. Insert or delete bases in DNA.
**sgRNA—single-guide RNA**	Consists of crRNA and tracrRNA, can direct Cas9-mediated genome editing.
**PAM**	Proto-spacer adjacent motifs on crRNA are particularly required and recognized by Cas9 protein.
**crRNA**	One CRISPR RNA base pair of the guide RNA in CRISPR/Cas9.
**tracrRNA**	Trans-activating chimeric RNA. Another CRISPR RNA base pair in CRISSPR/Cas9. It can promote crRNA processing.
**Lentiviral Vectors (LVs)—Lentiviruses**	The replication-defective retroviruses that are applied to introduce the target gene *in vivo* or *in vitro* for genome editing.
**Adenoviral Vectors (AdVs)—Adenoviruses**	The replication-defective viruses belong to the family of Adenoviridae. They are always used to carry the designed gene into targeted tissues or cells *in vivo* or *in vitro* for genome editing.
**Adeno-associated viral Vectors (AAVs)—Adeno-associated viruses**	The commonly used delivery vector in genome editing technologies for its potential site-specific integration ability and low immunogenic characteristics. They are icosahedral non-enveloped viruses in *Dependovirus* genus of *Parvoviridae* family.
**Liposomes**	Lipid made vesicles which can encapsulate designed DNA or RNA, and carry them into target tissues and cells by fusing to the cell membrane for genome editing *in vivo* or *in vitro*.
**Polymer**	Co-polymers made vesicles which can encapsulate both genes and proteins, and form particles with different sizes. They can also be utilized for designed genes or proteins delivery *in vivo* or *in vitro* to achieve gene editing therapy.
**Cell-penetrating peptides (CPP)**	Small peptides which can combine with target genes or proteins, and translocate across cell membranes to achieve the delivery and genome editing *in vivo* or *in vitro*.
